# Socio-demographic characteristics and cognitive performance in oldest old subjects asking for driving license renewal

**DOI:** 10.1186/s12877-020-01637-1

**Published:** 2020-07-11

**Authors:** Giuseppina Bernardelli, Palmina Caruso, Guido Travaini, Isabella Merzagora, Francesca Gualdi, Raffaela D. G. Sartori, Daniela Mari, Matteo Cesari, Valeria Edefonti

**Affiliations:** 1 Dipartimento di Scienze Cliniche e di Comunità, Università degli Studi di Milano, Milan, Italy; 2 Dipartimento di Scienze Biomediche per la Salute, Università degli Studi di Milano, Milan, Italy; 3 Facoltà di Medicina, Università Vita e Salute San Raffaele, Milan, Italy; 4 Università degli Studi di Milano, Milan, Italy; 5 Fondazione IRCCS Ca’ Granda Ospedale Maggiore Policlinico, Milan, Italy; 6 Geriatric Unit, Fondazione IRCCS Ca’ Granda Ospedale Maggiore Policlinico, Milan, Italy; 7 Branch of Medical Statistics, Biometry and Epidemiology “G. A. Maccacaro”, Department of Clinical Sciences and Community Health, Università degli Studi di Milano, via Venezian 1, 20133 Milan, Italy

**Keywords:** Age, Driving license renewal, Education, MMSE, MOCA, Oldest old subjects, Sex, Socio-demographic factors

## Abstract

**Background:**

No papers have examined the relationship between socio-demographic characteristics and cognitive performance in oldest old subjects (i.e, > = 80 years old) asking for driving license renewal. We hypothesize that, even in this highly functioning population, age, sex, and education influence cognitive performance, expressed as total or single domain (raw) test scores. This research question allows to describe, identify, and preserve independence of subjects still able to drive safely.

**Methods:**

We examined cross-sectionally a cohort of > = 80 years old subjects (at enrollment) asking for driving license renewal in the Milan area, Italy, 2011–2017. The analysis was restricted to 3378 first and 863 second visits where individual’s cognitive performance was evaluated. According to the study protocol, the Mini Mental State Examination (MMSE) test was administered at the first visit for driving license renewal and the Montreal Cognitive Assessment (MoCA) test at the second visit, following an additional renewal request. Ordinary least squares regression models were fitted at either time points. In each model, we included age, sex, and education as independent variables, whereas the dependent variable was total or single domain score for either test. In total, we fitted 15 regression models to assess our research hypothesis.

**Results:**

The median subject in our sample reached the maximum scores on domains targeting operational and tactical abilities implied in safe driving, but had sub-optimal scores in the long-term memory domain included among the strategic abilities. In multiple models, being > = 87 (versus 80- < 86 years old) significantly decreased the mean total and memory scores of MMSE, but not those of the MoCA. Females (versus males) had significantly higher mean total and long-term memory scores of either tests, but not other domains. Mean total and single domain scores increased for increasing education levels for either tests, with increments for high school graduates being ~ 2 of those with (at most) a junior high school diploma.

**Conclusions:**

Sex and education, as well as age to a lesser extent, predict cognitive functioning in our oldest old population, thus confirming that concepts like cognitive reserve and successful ageing are valuable constructs in the identification of older subjects still able to drive.

## Background

Population ageing affects most of the industrialized countries. In Italy, life expectancy at birth is the second highest in the world, being 80.6 years for men and 85.3 years for women in 2018 [1].

The increase in life expectancy is related to an increase in age-related conditions, including cognitive impairment and dementia [2–4]. In older subjects, those conditions may depend on demographic characteristics, such as age, education, and sex. A close association between age and dementia has been reported, with a higher speed of cognitive decline generally found with increasing age [5]. In addition, previous studies have supported the key finding that a higher education level is a strong predictor of sustained cognitive function in old ages [6–8] and may protect against age-related decline [9–11]. Although no significant differences were found between men and women on verbal, spatial, or other cognitive abilities in one paper [12], sex-related differences have been identified in episodic memory [12], verbal memory [8], cognitive speed and memory tasks [13]: women outperformed men on these tasks, despite their generally lower level of formal education [8].

Due to the higher life expectancy, an increased number of older drivers has been recently registered [14]. In Italy, about 19% of all driving license holders are aged over 60 years and only 63% of the women (versus 85% of men) has a driving license, with the lowest percentages being in the south of Italy; in the north of Italy, the Lombardy region shows about 17% of all registered driving licenses, as to the more recent official report on the topic [15].

As compared to those < 65 years, older subjects have a higher risk of car accidents [16, 17]. In a meta-analysis [18] based on 62 studies on impairment and risk of injury accidents, male and female drivers aged 75 years or more had a relative risk of being involved in car accidents of about 3.1 and 3.25, respectively, as compared to the corresponding groups with the lowest accident risks (male drivers aged 45–54 and female drivers aged 35–54 years, respectively). In addition, as also confirmed in [16] on a per person-mile of travel basis, accident involvement rate was found to be a U-shaped function of driver age, both for men and for women: the relative risk decreased from the 16–19 years cohort until it reached its bottom for drivers aged 45–54 and consistently increased for all the older age cohorts [18].

However, chronological age may be a crude criterion to prohibit driving. Driving allows independence, prevents social isolation and, on this way, provides a higher quality of life [19, 20]. In addition, when measures are taken per capita or per number of drivers, older drivers cause fewer crashes compared to younger age groups [16, 21]. Similarly, there has been a long-recognized association between extent of driving and crash involvement at any age group: the lower the annual mileage driven, the higher the per-distance crash rate. Because older drivers generally drive less distance per year than others, this association has been used to explain much of their apparent over-involvement in crashes [22]. For instance, in a Dutch travel survey, when crash rates were compared after being matched for yearly driving distance, most drivers aged 75 years and above were indicatively safer than all other drivers, with only those travelling less than 3000 km/year (~ 10% of all older drivers in the survey) being at elevated crash rates [22].

It is, therefore, essential to develop suitable criteria for identifying older subjects still able to drive. One conceivable possibility is that older drivers will be tested based on their physical and neuropsychological abilities [23, 24]. The British Psychological Society [25] has suggested to assess the following cognitive and psychological functions: perception, executive (or frontal) functions (e.g. plan, anticipate and make decisions, practical skills), language, memory, and personality characteristics (e.g. self-control, emotional stability, and social responsibility). With additional details, Wagner and colleagues (2011) [21] highlighted that driving requires the integration of vision, motor, and high-level cognitive functions, including visual attention, visual perception, executive function, as well as the following three dimensions of long-term memory: episodic, semantic, and procedural memory (for detailed definitions of the single cognitive functions, see **eTable** **1**). A similar indication has come from two papers [26, 27] which posed self-regulated driving abilities within the context of the “Michon model” (1985) [28] and distinguished the three hierarchical levels of cognitive control of driving into operational, tactical, and strategic levels. In detail, the operational level implies automatic operations typically related to driving, the tactical level deals with modifications of the driving behaviour for adapting to changes in the environment, and strategic behaviors refer to trip planning, including decisions to avoid dangerous situations (for detailed definitions of hierarchical levels, see **eTable** **2**).

Among the available tests targeting the previous domains, the Mini Mental State Examination (MMSE) test [29] has been suggested for the assessment of cognitive abilities related to a safe driving [30]. Although it is widely used as a brief screening instrument for dementia and as a proxy for Alzheimer’s Disease staging [31, 32], it is not sensitive enough to distinguish mild cognitive impairment (MCI) subjects from healthy ones [33, 34]. The Montreal Cognitive Assessment (MoCA) [35] can predict dementia in people with MCI, and, because it tests for executive function, it is useful for subjects with normal MMSE scores. Nonetheless, both tests are able to verify the presence of behavioral and neuropsychological signs of cortical dementia (impaired memory, orientation, and language functions) and of subcortical dementia, where slow movement and loss of attention are observed [36]. To our knowledge, only 2 papers have compared the 2 tests in terms of driving performance. The former one [37] concluded that they similarly predicted driving test outcomes. The latter one [38] indicated that MoCA reliably identified at-risk individuals who had a pre-existing diagnosis of cognitive impairment, but not those without diagnosis; in addition, no significant results were found for MMSE in either sample.

Upon request of the Agency for Health Protection of Milan (Agenzia di Tutela della Salute della Città Metropolitana di Milano - ATS Milano), Milan, Italy, the University of Milan is currently carrying out a neuropsychological screening assessment of oldest old subjects [i.e., subjects > = 80 years [39, 40]] who would like to renew their driving license and are sent there for evaluation by the Local Medical Commission in charge of license renewals. Since 2011, cognitive and psychomotor functions have been assessed through the MMSE test (odd visits) and the MoCA test (even visits), whenever subjects ask for additional renewals. Although collected with a different aim, this information provides an overall picture of the cognitive status, and its determinants, of a very interesting subpopulation of oldest old subjects.

The main objective of this paper is to assess if total and single domain scores of either test depend on socio-demographic characteristics, including age, sex, and education. Within this highly selected and functioning subpopulation of Italian oldest old subjects, we hypothesize that:

1. Regardless of the test used, age, sex, and education will still influence *total cognitive performance*. In detail, we expect that cognitive performance will: 1.a decrease with increasing age; 1.b increase with increasing levels of education; 1.c not depend on sex;

2. Regardless of the test used, age, sex, and education may affect the *single cognitive domains under investigation*. In detail, we expect that visuospatial and time orientation, memory, attention and calculation, language, and executing function will: 2.a worsen with increasing age; 2.b be higher with higher levels of education; in addition, we expect that: 2.c women will perform better than men in language and long-term memory tasks;

3. Results will be different when MoCA, instead of MMSE, test is considered. In detail, we expect MMSE scores to be higher than MOCA ones, at the overall and single-domain levels.

## Methods

### Study design

In the following, we provide details on study design and characteristics, following the guidelines included in “REporting of studies Conducted using the Observational Routinely collected health Data” (RECORD) statement [41], which concern routinely collected health data, obtained for administrative and clinical purposes without specific a priori research goals. Since May 2011, one in a group of expert psychologists from the Legal Medicine Section of the University of Milan filled in a paper-and-pencil brief anamnesis and cognitive performance test for any subjects > = 80 years sent there for evaluation within a broader expert-based program of assessment of cognitive performance of oldest old subjects asking the Local Medical Commission of the ATS Milano for driving license renewal. In accomplishment of the more recent Italian regulation on the topic (Law 120 “Provisions on road safety”, 2010), the program was aimed at integrating standard information on general health status, disabilities, and comorbidities with cognitive performance, to provide a more comprehensive evaluation of driving abilities in oldest old subjects. The final decision on driving license renewals is in charge of the Local Medical Commission, ATS Milano. Similarly, at any driving license expiration and subsequent renewal request, the Local Medical Commission asks subjects > = 80 years old to carry out the necessary cognitive performance re-evaluation, as long as the other necessary general health requirements are still present.

### Selection of variables

At each visit, an expert psychologist asked subjects to provide information on age, date of birth, sex, and years of education. Date of test administration and type of test administered (MMSE or MoCA) were also recorded. The interviewer recorded single domain scores and summed up scores to get the total (raw) score. Results were reviewed at a later stage.

The study protocol indicated that the psychologist had to administer the MMSE test at the first visit and the MoCA at the second visit (at a minimum distance of ~ 6 months); similarly, the MMSE test was administered at the next odd visits and the MOCA at the next even visits. This decision reflected both the different characteristics of the two tests and the absence of any evidence from the literature on cognitive performance and driving abilities at the time of protocol drafting; in addition, subjects may remember test questions from one renewal to the next one and a change in the administered test would avoid a learning effect to be present in successive administrations. In **Additional file****1****– Materials and methods - Selection of variables**, we provided details on total and single domain scores, as well as domain definitions and names used in the current paper to improve comparability between test domains. In addition, we provided in **eTable** **2** a cross-classification of cognitive functions, as measured by the MMSE or MoCA tests, in terms of the three hierarchical levels of cognitive control of driving suggested within the Michon model [28]. Briefly, the total score for both tests ranges from 0 to 30, a MMSE score > =24 or a MoCA > = 26 indicate normal cognitive functions. In addition, within the Michon model, for both tests: 1. visuospatial orientation and constructive apraxia contributed to the more elementary operational level of driving abilities; 2. attention and calculation supported the tactical level; 3. long-term memory and language targeted the more difficult strategic level (see domains in italics in **eTable** **2**). Some domains were assessed in one test only, including short-term memory in MMSE, which contributed both to the tactical and to the strategic level. For the operational level, both MMSE and MoCA scores can assume values from 0 to 11, for the tactical level, the MMSE range was 0–8, and the MoCA range was 0–6, for the strategic level, the MMSE range was 0–14, and the MoCA range was 0–13.

The protocol has been approved by the suitably constituted Ethics Committee of the University of Milan (see the Declarations section for details) within a broader research project and it conforms to the provisions of the Declaration of Helsinki (as revised in Brazil 2013). Subjects have given written informed consent to participate and patient anonymity has been preserved.

### Selection of subjects

We processed 4768 of the 4840 driving license renewal visits carried out at the University of Milan from May 2011 to March 2017 (reason for exclusion: subject < 80 years old). Inconsistencies in visit information were corrected (see **Additional file****1****– Materials and methods - Selection of subjects** and **eTable** **3**). Overall, there were 3392 (71.14%) first visits, 945 (19.82%) second visits, 307 (6.44%) third visits, 92 (1.93%) fourth visits, 29 (0.61%) fifth visits, 2 (0.04%) sixth visits, and only 1 (0.02%) seventh visit.

In the current paper, we based our analysis on 4241 first and second visits, including 3378 first visits where the MMSE test was administered (99.59% of all first visits, 89.74% of all MMSE tests administered) and 863 second visits where the MoCA was administered (91.32% of all second visits, 85.96% of all MoCA tests administered) (reasons for exclusion: in 14 first visits MoCA was administered and in 82 second visits MMSE was administered, see **eTable** **3****).**

### Statistical analysis

Ordinary least-squares linear regression models were used to assess the relationship between socio-demographic characteristics and total (or single domain) scores, with test score being the dependent variable, and socio-demographic characteristics the independent variables (see **Additional file****1****– Materials and Methods - Statistical analysis**). For each test, we ran simple and multiple linear regression models of increasing complexity, including two- and three-way interactions models. Model selection was carried out through likelihood ratio tests. *P*-values were two-sided and significance was assumed when the *p*-values were less than 0.05. Calculations were carried out using the open-source statistical computing environment R [42].

## Results

### Sample description: socio-demographic characteristics, total, and single domain scores (hypothesis 3)

Table 1 shows the distribution of the socio-demographic characteristics stratified for those subjects who received the MMSE at the first visit (left part) and the MoCA at the second visit (right part). A non-negligible proportion of subjects of 90 years or more (~ 9% for MMSE and 23% for MoCA) was present in either group. About 90% of the subjects were males in either group, the percentage being slightly lower with MoCA. About 60% of the subjects had a high education level in either group, with ~ 30% of subjects who attended or finished the university in each group.

**Table 1:** Distribution (%) of socio-demographic characteristics of the sample within the cross-sectional analyses of the Mini Mental State Examination test scores and the Montreal Cognitive Assessment test scores

	MMSE test score (first visit)		MoCA test score (second visit)	
	**N**	**%**	**N**	**%**
	3378	100.00	863	100.00
**Socio-demographic characteristic**				
**Age (years)**^a^				
[80, 86)^b^	1246	36.89	26	3.01
86	830	24.57	183	21.21
87	470	13.91	200	23.17
[88, 90)^b^	515	15.25	259	30.01
[90, 99)^b^	317	9.38	195	22.60
**Sex**				
Female	366	10.83	70	8.11
Male	3012	89.17	793	91.89
**Education**				
≤ Primary school	744	22.02	161	18.66
≤ Junior high school	690	20.43	163	18.89
≤ High school graduate	1016	30.08	278	32.21
> High school graduate	928	27.47	261	30.24

ABBREVIATIONS: *MMSE* Mini Mental State Examination test; *MoCA* Montreal Cognitive Assessment test

^a^The same age categories were chosen for the analysis of MMSE and MoCA test scores. At either visit occasion, the chosen categories allowed to: 1. guarantee the minimum frequency of 3% per cell and 2. distribute cell frequencies as much as possible across age categories for a fixed visit occasion

^b^For each age category indicated with brackets (e.g., [80, 86)), the square bracket indicated that the lower value (e.g., 80) was included in the interval, the curved bracket indicated that the upper value (e.g., 86) was excluded from the interval

**eTable** **4** shows descriptive statistics representing the distribution of total and single domain scores for the MMSE and MoCA tests in our population (see **Additional file****1****– Results**). Briefly, with the exception of long-term memory, 75% (MMSE) and 50% (MoCA) of the sample reached the maximum value of the single domain scores. In addition, the minimum value for the total MMSE test score was 17, as compared to 7 for MoCA. The median performance of the oldest old subjects under cognitive evaluation was 29 for MMSE and 25 for the MoCA test. Given the cut offs for preserved cognitive functioning of 24/30 for the MMSE and 26/30 for the MoCA, our population included more subjects with preserved cognitive functions according to the MMSE (> 75% of the sample) than to the MoCA (> 25- < 50% of the sample). Most of the difference on the median scores was due to the long-term memory domain, where subjects ranked 2/5 in the MoCA test and 2/3 in the MMSE test.

In addition, Table 2 reports the MMSE and MoCA scores reached by the median subjects for each of the three hierarchical levels of the Michon model. At the operational and tactical levels, the median subject reached the maximum (operational level: 11 points for both tests; tactical level: 8 points for MMSE and 6 for MoCA), whereas, at the strategic level, the median subject received 13 out of 14 points for MMSE and 10 out of 13 points for the MoCA. In both cases, the maximum was not reached due to long-term memory domain, where the median subject lost 1 MMSE and 3 MoCA points.

**Table 2:** Mini Mental State Examination and Montreal Cognitive Assessment test scores for the median subject in our sample, within each of the three hierarchical levels of cognitive control of driving suggested within the Michon model [28]

		Driver Behavior^a,b,c^	
	**Strategic Level**	**Tactical Level**	**Operational Level**
**Driving ability**	Driving with a copilot, planning the trip before driving, hitting the traffic, avoiding dangerous driving situations	Choosing direction, speed adaptation, anticipatory behavior	Steering, braking, using the vehicle controls, shifting gears
**Cognitive functions measured by MMSE (range of possible score values)**	Short-term memory^a^*(0–3)* *Long-term memory (0–3)* *Language (0–8)*	Short-term memory^a^*(0–3)* *Attention and calculation (0–5)*	*Visuospatial orientation (0–10)* *Constructive apraxia (0–1)*
**MMSE score for the median subject over the maximum score**	13/14	8/8	11/11
**Cognitive functions measured by MoCA (range of possible score values)**	*Long-term memory (0–5)* Abstraction^b^*(0–2)* *Language (0–6)*	*Attention and calculation (0–6)*	*Visuospatial orientation (0–6)* Visuospatial functioning^b^*(0–3)* *Constructive apraxia (0–2)*
**MoCA score for the median subject**	10/13	6/6	11/11

ABBREVIATIONS: *MMSE* Mini Mental State Examination test; *MoCA* Montreal Cognitive Assessment test

^a^Domains available in the MMSE test only

^b^Domains available in the MoCA test only

^c^Domains common to both tests were indicated in italics

Table 3 and Table 4 provide results from the multiple regression analyses assessing the effect of each socio-demographic characteristic on total (or single domain) (raw) test scores, after adjusting for the two-remaining socio-demographic characteristics. There is, indeed, no evidence in favor of adding either two or three-way interactions among the socio-demographic characteristics in the multiple models including all the three socio-demographic characteristics (*p*-values from likelihood ratio tests > 0.01 for any comparison tested, data not shown).

**Table 3:** Point estimates, standard errors (in parenthesis), and *p*-values^a^ from ordinary least-squares multiple regression models on total (or single domain)^b^ score of the Mini Mental State Examination test (*N* = 3378)

				MMSE test score			
	Visuospatial orientation (0–10)	Short-term memory (0–3)	Attention and calculation (0–5)	Long-term memory (0–3)	Language (0–8)	Constructive apraxia (0–1)	Total (raw) score (0–30)
**Socio-demographic characteristic**^c^							
**Intercept**	9.621 (0.049)***	2.954 (0.013)***	4.594 (0.038)***	1.984 (0.065)***	7.534 (0.031)***	0.854 (0.016)***	27.553 (0.121)***
**Age (years)**							
86	0.038 (0.032)	− 0.07 (0.009)	0.029 (0.025)	0.033 (0.043)	0.016 (0.021)	0.001 (0.010)	0.102 (0.080)
87	0.070 (0.039)^.^	−0.003 (0.010)	0.035 (0.030)	0.101 (0.052)^.^	0.016 (0.025)	0.016 (0.013)	0.214 (0.097)*
[88, 90)	−0.051 (0.038)	− 0.038 (0.010)***	0.008 (0.030)	−0.104 (0.050)*	− 0.024 (0.024)	0.005 (0.012)	− 0.197 (0.094)*
[90, 99)	−0.104 (0.046)*	− 0.001 (0.012)	− 0.014 (0.035)	− 0.233 (0.060)***	−0.003 (0.029)	0.009 (0.015)	−0.351 (0.113)**
**Sex -– Male**	−0.030 (0.040)	−0.010 (0.011)	− 0.015 (0.031)	−0.219 (0.053)***	− 0.018 (0.026)	−0.017 (0.013)	− 0.308 (0.100)**
**Education**							
≤ Junior high school	0.068 (0.038)^.^	0.042 (0.010)***	0.240 (0.030)***	0.197 (0.051)***	0.245 (0.025)***	0.061 (0.012)***	0.842 (0.095)***
≤ High school graduate	0.185 (0.035)***	0.047 (0.009)***	0.358 (0.027)***	0.334 (0.046)***	0.356 (0.023)***	0.116 (0.011)***	1.400 (0.087)***
> High school graduate	0.178 (0.036)***	0.046 (0.009)***	0.364 (0.028)***	0.406 (0.047)***	0.396 (0.023)***	0.133 (0.012)***	1.516 (0.089)***

ABBREVIATIONS: *MMSE* Mini Mental State Examination test

^a^Significance codes for the *p*-values: 0 ‘***’ 0.001 ‘**’ 0.01 ‘*’ 0.05 ‘^.^’ 0.1 ‘’ 1

^b^Domains were presented in the order in which the MMSE test assessed them

^c^The reference categories for each socio-demographic characteristic included in the models were as follows:

Age: [80, 86); Sex: Female; Education: ≤Primary school. For each age category indicated with brackets, the square bracket indicated that the lower value (e.g., 80) was included in the interval, the curved bracket indicated that the upper value (e.g., 86) was excluded from the interval. For details on how age categories were chosen, see the footnote in Table 1

**Table 4:** Point estimates, standard errors (in parenthesis), and *p*-values^a^ from ordinary least-squares multiple regression models on total (or single domain)^b^ score of the Montreal Cognitive Assessment test (*N* = 863)

				MoCA test score				
	Visuospatial orientation (0–6)	Attention and calculation (0–6)	Long-term memory (0–5)	Language (0–6)	Constructive apraxia (0–2)	Visuospatial functioning (0–3)	Abstraction (0–2)	Total (raw) score (0–30)
**Socio-demographic characteristic**^c^								
**Intercept**	5.783 (0.103)***	4.908 (0.214)***	2.116 (0.368)***	4.771 (0.186)***	0.813 (0.140)***	2.322 (0.149)***	1.368 (0.112)***	22.172 (0.686)***
**Age (years)**								
86	0.060 (0.091)	0.045 (0.189)	0.082 (0.325)	0.109 (0.164)	−0.057 (0.123)	0.143 (0.132)	−0.041 (0.099)	0.360 (0.650)
87	0.063 (0.091)	0.058 (0.188)	−0.061 (0.323)	−0.003 (0.163)	0.066 (0.123)	0.174 (0.131)	0.043 (0.098)	0.274 (0.603)
[88, 90)	−0.003 (0.090)	0.007 (0.185)	−0.132 (0.319)	−0.011 (0.161)	0.044 (0.121)	0.138 (0.130)	−0.021 (0.097)	0.011 (0.595)
[90, 99)	−0.035 (0.091)	0.117 (0.188)	−0.156 (0.324)	0.051 (0.164)	0.075 (0.123)	0.162 (0.132)	−0.059 (0.099)	0.194 (0.604)
**Sex - Male**	0.074 (0.054)	−0.166 (0.112)	−0.535 (0.193)**	0.014 (0.097)	0.041 (0.073)	−0.060 (0.078)	0.028 (0.059)	−0.711 (0.359)*
**Education**								
≤ Junior high school	0.001 (0.048)	0.526 (0.100)***	0.336 (0.172)^.^	0.419 (0.087)***	0.495 (0.065)***	0.198 (0.070)**	0.301 (0.052)***	2.215 (0.321)***
≤ High school graduate	0.039 (0.043)	0.753 (0.089)***	0.518 (0.153)***	0.672 (0.077)***	0.870 (0.058)***	0.296 (0.062)***	0.408 (0.047)***	3.578 (0.286)***
> High school graduate	0.070 (0.044)	0.860 (0.090)***	0.737 (0.155)***	0.803 (0.078)***	0.888 (0.059)***	0.267 (0.063)***	0.524 (0.047)***	4.195 (0.290)***

ABBREVIATIONS: *MoCA* Montreal Cognitive Assessment test

^a^Significance codes for the *p*-values: 0 ‘***’ 0.001 ‘**’ 0.01 ‘*’ 0.05 ‘^.^’ 0.1 ‘’ 1

^b^Domains were presented in the order in which the MMSE test assessed them

^c^The reference categories for each socio-demographic characteristic included in the models were as follows:

Age: [80, 86); Sex: Female; Education: ≤Primary school. For each age category indicated with brackets, the square bracket indicated that the lower value (e.g., 80) was included in the interval, the curved bracket indicated that the upper value (e.g., 86) was excluded from the interval. For details on how age categories were chosen, see the footnote in Table 1

### Determinants of mini mental state examination total score (hypothesis 1 - MMSE)

Table 3 presents results on the total and single domain MMSE test scores. The MMSE total score significantly depended on age, sex, and education. The mean test score for the subjects in the reference category [i.e. [80–86) years old females with up to the fifth grade of primary school, where 80 was included and 86 excluded from the interval indicated as [80–86) years] was about 27.5 [testing the null hypothesis of model intercept (i.e. mean total score) being equal to 0: *p*-value< 0.001]. Having 87 or more years of age significantly decreased the mean test score of about 0.20 [e.g. for the [88,90) category, *p*-value< 0.05] and 0.35 [for the [90,99) category, *p*-value< 0.01], as compared to being [80–86) years old subjects. Similarly, as compared to females, males significantly decreased their total score of about 0.31 (*p*-value< 0.01). Higher levels of education significantly increased the mean total score of about 0.85 (≤ Junior high school, *p*-value< 0.001), 1.40 (≤High school graduate, *p*-value< 0.001), and 1.52 (>High school graduate, *p*-value< 0.001), as compared to have up to the fifth grade of primary school. The increment in the test score was almost the double (i.e. from 0.85 to 1.52) going from the “≤ Junior high school” to the “> High school graduate” category of education.

### Determinants of single domain scores for the mini mental state examination test (hypothesis 2 - MMSE)

Results for the single domain analysis showed similarities with those of the total score analysis. The mean single domain scores were extremely high, with the partial exception of long-term memory (~ 2 out of 3); all the mean scores for the single domains were significantly different from 0 (see the “Intercept” row in Table 3, all *p*-values< 0.001). The highest categories of age were significantly and negatively related to short-term (about − 0.040 for the [88,90) year category only, *p*-value< 0.001) and long-term memory (about − 0.10 for the [88,90) and − 0.25 for the [90,99) categories of age, *p*-values< 0.05 and < 0.001, respectively). As compared to females, males had lower mean single domain scores, but differed in a significant way only when long-term memory was assessed (*p*-value< 0.001). The two highest levels of education (“≤ Junior high school” and “≤High school graduate”) consistently and significantly doubled their positive effect on single domain scores (all *p*-values < 0.001 across domains for these two categories), as compared to the “≤ Junior high school” education category; the short-term memory domain had, however, a positive effect that was stable across education categories (of about 0.04–0.05, both *p*-values < 0.001).

### Determinants of the Montreal cognitive assessment total score (hypothesis 1 - MoCA)

Table 4 presented results on the total and single domain MoCA test scores. The MoCA total score significantly depended on sex and education. The mean test score for a [80–86) (i.e. 80 included, 86 excluded from the interval) years old female with up to the fifth grade of primary school was about 22.20 (*p*-value< 0.001). Similarly, being a male significantly decreased the total score of about 0.70 (*p*-value< 0.05), as compared to being a female. Higher levels of education significantly increased the mean total score of 2.22 (≤ Junior high school, *p*-value< 0.001), 3.60 (≤High school graduate, *p*-value< 0.001), and 4.20 (>High school graduate, *p*-value< 0.001), as compared to being up to primary school graduate students. The trend was similar to that of the MMSE test, with the effect estimate of the “>High school graduate” category being almost double the effect of the “≤ Junior high school” category.

### Determinants of single domain scores for the Montreal cognitive assessment test (hypothesis 2 - MoCA)

Results for the single domain analysis showed similarities with those of the total score analysis. No significant effect was found for age at any category. Being a male significantly decreased the long-term memory domain score (point estimate ~ − 0.55, *p*-value< 0.01), but not any other one. Finally, except for the visuospatial orientation domain (nonsignificant *p*-value), higher levels of education were still significantly and positively related to the single domain scores, with a strong and positive trend of a double effect estimates in the “>High school graduate”, as compared to the “≤ Junior high school” category (all *p*-values< 0.001 across the mentioned categories and single domains). Similar results were observed when simple regression models were considered.

## Discussion

The present study considers a convenience sample of oldest old persons (i.e. those with 80 years of age or more) from the metropolitan area of Milan, Lombardy, Italy, and assesses the role of age, sex, and education in influencing their cognitive performance, as measured cross-sectionally by either the MMSE or the MoCA tests. Our findings suggest that sex and education, as well as age to a lesser extent, are strong predictors of preserved cognitive functions, even in this highly functioning oldest old population, where, in median, domains targeting operational and tactical driving abilities were reached. Domains targeting strategic abilities were not fully met, due to sub-optimal performances in long-term memory domains for both tests.

### Determinants of total test scores (hypothesis 1)

Concerning hypothesis 1.a, we confirm that age is a significant predictor of the mean total performance for the MMSE, but not for the MoCA test [5, 43–47]. Four factors dealing with the presence of preserved driving abilities in older subjects are of great relevance [18]. First, the health of the drivers: as they become older, reaction time worsens and the ability to drive safely is likely reduced. Second, morbidity and use of medicines in general increase with age. Third, at older ages the probability that a driver has dementia increases [48, 49] and cognitive impairment also means reduced ability to process information and make decisions. Forth, older drivers have likely increased frailty, making them more susceptible to serious injury. With its negative significant relationship with age for subjects aged 87 years or more, the less sensitive MMSE test [21] could have captured one or more of these aspects. Notably, although the relationship with age was stronger for increasing age categories, the effect was still moderate and of modest statistical significance; this may allow to speculate that, although oldest old subjects become older over time, the impact of ageing on cognitive performance was globally modest in our sample of highly functioning subjects.

A failure in finding a significant effect of age on MCI-targeted MoCA test may be similarly interpreted. Despite likely reduced levels of physical functioning - which are the main reasons to be evaluated by the Local Medical Commission - our oldest old subjects were mainly in good mental health at visit occasions. When compared with a subset of 48 80+ years old Italian healthy subjects from the first normative study on the MoCA test as translated into Italian (including 415 subjects from different Italian districts, representing the Italian healthy population, and with a mean raw MMSE score of 28) [50], the mean raw MoCA score in our sample was 24.54 (see **Additional file****1****– eTable 4**) versus 17.8 from the 80+ years old subjects from the normative study; however, only 10% of the 80+ years old subjects interviewed in the normative study had 13 or more years of education versus 32% in our sample, so we cannot exclude that the higher education level in our sample is responsible in part for our higher mean score. These latter observations supports the hypothesis of the high resilience and ability to adapt to age-associated challenges of our subjects, independently from being 80- or 90-years old or more [51].

In conclusion, although age itself is a proxy of several other aspects related to driving abilities, it should not necessarily be used to assume driving capability or inability; however, it’s still possible that age acts more strongly on some specific domain scores, even though not on all of them.

Moreover, we confirm the key role of education in increasing mean test scores (hypothesis 1.b). No matter of the test under investigation, education showed a consistent trend, where increasing levels of education provided increasing total scores in both tests. More years of education and life-time participation in intellectual activities contribute to the “cognitive reserve”, here intended as the ability to cope with higher levels of brain damage without presenting clinical symptoms of dementia [52]. In addition, lifestyle is a key component in the pathway including education and cognitive reserve. An active involvement in hobbies, outdoor and social activities prevents loneliness and social isolation; at the extreme consequences, it also contributes to an increase of the brain functioning against mental decline [52, 53]. On the other hand, driving license itself is a key component of an independent lifestyle and contributes to maintain good cognitive functions as age increases. In conclusion, the highest the education level of the subject the highest should be the probability that he/she still has preserved driving abilities.

Contrary to hypothesis 1.c, in our sample we found that sex is a significant predictor of the total test score, with females having a higher mean total score in both tests [54]. As compared to males, females in general may have lower levels of age-associated cognitive decline, possibly because of biological mechanisms such as the effects of estrogen or sex-specific cognitive reserve, but also due to sociocultural factors, as emerged for Whites – including a cohort in the neiborhood of Milan - in an updated pooled analysis of individual-level longitudinal data from 20 population-based cohorts (2–15 years of follow-up) from 15 countries over 5 continents, including 48,522 individuals (58.4% women) aged 54–105 (mean = 72.7) years and without dementia at baseline [55].

Based on the neuropsychological evaluation, women should be more likely than men to receive license renewals at the same general health conditions. Evidence from a meta-analysis on 62 studies suggested that, when compared to the lowest risk categories of the same sex, male and female drivers aged 75+ had a similar relative risk of injury of ~ 3 [18]; however, when the analysis was restricted to the 9 studies assessing the relationship between age and gender of car drivers and their involvement in injury accidents, 75+ years-old females showed the second highest relative risk of being involved in injury accidents, immediately following the 16–19 years old males, with a relative risk over 5 versus 3 for men aged 75+ [18]. Although women are generally considered to be more careful drivers than men, and are charged for traffic offences much less often than men, the following aspects should be taken into account. Firstly, females usually drive less than men (e.g. [56, 57]) and the accident involvement rate per kilometre of driving decreases as driving distances increase. In detail, Italian female drivers (63% of all Italian women) of any age drove 10% less than men in 2016 (women: 10600 km/year; men: 11500 km/year, from the Italian website (in Italian) https://www.scuolaguida.it/it/Statistiche/art/2232-guidano-meglio-gli-uomini-o-le-donne/, date last access: June 17, 2020) and we expect this difference to be definitely higher for older women, although we were not able to find out Italian data on this aspect. Secondly, women tend to drive smaller cars than men (e.g. [18, 56]), with likely not as good protection against injury in an accident as larger cars. Thirdly, women tend to drive more in cities (e.g. [57]), where the risk of accidents is higher than in rural areas. All the mentioned aspects fit perfectly with our sample of oldest old drivers in the urban area of Milan, with its 1352 millions of citizens in 2017.

### Determinants of single domain scores (hypothesis 2)

Concerning hypothesis 2.a, age was found to be a significant predictor of the long-term memory domain in the MMSE test, but not in the MoCA. The former finding has been previously supported in the literature [5], whereas the latter one may be attributed to the different scoring system of the long-term memory domain in the two tests (see the Results section, paragraph 3.1). Indeed, although both tests ask to recall words within approximately 5 min, the MoCA considered 5 words, as compared to the 3 of MMSE, in accordance with its main objective of targeting subjects with MCI.

We also confirm hypothesis 2.b on the key role of education in increasing single domain scores of both tests, with the only exception of visuospatial orientation in MoCA. This is in line with previous literature [8] on the role of age, sex, and education on executive functioning, verbal fluency, verbal memory, and cognitive speed tasks in healthy 64–81 years old subjects from the Maastricht Aging Study.

A similar indication has indirectly come from the literature examining risk factors for Alzheimer’s disease and other dementias (e.g. [58, 59]), where low education was found to be a risk factor. In detail, based on a meta-analysis of ~ 20 studies up to 2005, the relative risks for low versus high education level were significantly higher for Alzheimer’s disease and for all dementias. In addition, the estimated population-attributable risk of Alzheimer’s disease worldwide was the highest for low educational attainment, as compared to diabetes, midlife hypertension, midlife obesity, physical inactivity, depression, and smoking [59]. Within the “cognitive reserve” hypothesis, a higher level of education can delay the clinical expression of the disease in subjects with brain damage. Indeed, education provides strategies to solve the cognitive requirements and modifies neural connectivity and plasticity [58].

Finally, models for successful ageing (e.g. Rowe and Kahn’s (1987, 1997) model [60, 61], where avoidance of disease and disability, maintenance of high physical and cognitive function, and sustained engagement in social and productive activities were acknowledged) further suggest that higher levels of education support the good functioning of oldest old subjects. Indeed, the percentage of adults aging successfully increases markedly with increasing level of education (e.g. [62–64]). In detail, models for estimating the prevalence of successful agers within and across countries (e.g. [62, 64]) and over time (e.g. [62]) provide control for age, sex, and education, as well as racial-ethnic background and socio-economic status, possibly at the individual level. In one of the mentioned studies on retired older adults from the US [63], after adjusting for the previous covariates, the odds of aging successfully increased substantially for those with higher levels of education, income, and wealth. Notably, significant differences remained for each indicator of socio-economic status after simultaneously controlling for the others [63]. This suggests parallel roles of each socio-economic component on successful aging. For instance, whereas higher income allows greater access to health promoting resources, the cognitive resources (e.g. capacity for problem solving, or knowledge) garnered through higher education may foster a sense of control that results in better health practices [65].

Similarly, each socio-economic variable may show a positive relationship with purpose in life. High levels of income and education, as well as high professional status, may be a direct source of purpose in life; they provide more knowledge about how to reach desired goals more effectively, reflect success in life, and contribute to the perception of one’s past life as successful and meaningful. There are also indirect associations between socioeconomic status and purpose in life, mediated through activities and attainments. For example, education gives access to a broad range of interests and meaningful activities; ample financial resources enable individuals to engage in a greater variety of activities that may contribute to purpose in life [66].

Concerning the effect of sex on single domains, we have speculated that females would have performed better in language and long-term memory tasks than males. In our sample, sex was found to be a significant predictor of long-term memory, but not of language. In addition, we can argue that previous findings [8, 54] on better performance of women on verbal memory tasks (but not on other cognitive domains, such as speed of information processing and attention) are related to ours, as most of the memory tasks rely on verbal abilities [12].

Finally, when relating results on single domain scores to driving abilities, we showed that a median subject asking for driving license renewal reached the maximum MMSE or MoCA scores in the (easiest) operational and tactical levels indicated in the Michon model, but failed in doing that at the more complex strategic level; this failure was due to a sub-optimal performance in the long-term memory domain. All the previous observations taken together provided support to the following findings:

1. As a proxy for more complex abilities required for a safe driving, long-term memory should receive a higher weight in the evaluation of cognitive performance in driving license renewals;
2. A preserved long-term memory is more likely to be present when relatively younger females with the highest level of education are interviewed;
3. After confirmation of education being the more consistent and strong determinant of cognitive function at the single domain level, interviews on driving license renewals should always ask about the highest level of education reached by the subject in his/her life.

### Comparison of results between mini mental state examination and Montreal cognitive assessment tests (hypothesis 3)

Finally, we have hypothesized that results would have been different in the 2 tests, with higher scores in the MMSE one (hypothesis 3). Indeed, in the regression models, we observed a higher mean total score in the reference category (i.e., [80–86) years old females with up to the fifth grade of primary school) with MMSE test (intercept: 27.6) as compared to MoCA (intercept: 22.2). This aspect likely reflects differences in test administration**:** MoCA is longer than MMSE to fill in and our subjects generally reported more difficulties in carrying out the single tests of the MoCA battery. However, both education and sex had a stronger effect when MoCA test was considered: the highest level of education (i.e., “>High school graduate”) added to the mean total score 4.2 points (versus 1.52 of the MMSE) and males showed a mean total score decreasing by 0.71 with MoCA (versus 0.31 with MMSE). This may be due to the fact that the MoCA total score has a reduced ceiling effect in comparison with the MMSE (3rd quartile of total score distribution: 30 for the MMSE versus 27 for the MoCA test), as also emerged for MCI and healthy controls in Trezpack et al. [67].

Both knowledge of the theory and the practical administration of the two tests to our sample would suggest that MoCA should perform better in capturing driving abilities (see for instance comments in [21]). However, literature comparing the two tests in terms of driving performance [37, 38] is still scanty and inconclusive. Indeed, the ability of MoCA to reliably identify at-risk individuals who had a pre-existing diagnosis of cognitive impairment was highly expected (e.g., [67]) and no significant results were found for the MMSE test in either sample [37, 38]. Within a public health perspective, where both traffic safety of the population and independence of oldest old subjects are major priorities, a fair evaluation of driving ability should integrate the neuropsychological assessment from both tests with practical sessions at driving simulators in a sufficiently large sample of subjects.

### Strengths and limitations of the study

Among study limitations, the major one is that our results have a limited generalizability. Indeed, we interviewed oldest old subjects who live in a metropolitan area of the industrialized northern Italy, have a very high level of education, and do not present severe cognitive impairment (minimum MMSE total score: 17; 1st quartile for MoCA score: 23), although a few MCI cases are likely to be present (2.5% percentile of MMSE total score: 23; minimum MoCA total score: 7). In addition, our sample included only ~ 10% of women. This sex ratio is, however, in line with existing data on Italian car drivers > 65 years of age, with > 80% of male drivers registered in 2008 [14]. We expect the sex ratio to change drastically in the next birth cohorts, with possibly different relationships with measures of cognitive impairment and memory too. We also lack of important individual-level information, including subject’s major diseases and treatments; in addition, we were not able to relate test performance to driving test outcomes or accidents [38]. Similarly, longitudinal analyses including either MMSE results from visits 1, 3, and 5 or MoCA results from visits 2, 4, and 6 could not be carried out so far, due to the limited number of subjects available at later visit occasions. Finally, our design did not allow for a formal comparison of MMSE and MoCA tests, as they were administered at different time points. However, we were able to cross-sectionally confirm some previous evidence on the higher sensitivity of MoCA test in identifying MCI cases [67].

Among study strengths, to our knowledge, this is the first Italian study that describes socio-demographic characteristics of subjects asking for driving license renewal and relates them to cognitive impairment. Our sample is large and it is expected to grow in future years, as far as there is a legal requirement for collecting information on cognitive functions of oldest old subjects asking for driving license renewal. This provided us enough power to analyze each test domain separately and to conclude that long-term memory is a critical domain to target as a proxy for more complex driving abilities, indicated as “strategic abilities” within the Michon model. Although there is no previous literature on such an Italian population, our results on total and single domain scores are generally sound.

## Conclusions

In conclusion, our work has shown that sex and education, as well as age to a lesser extent, are strong predictors of cognitive function, even in this highly functioning oldest old subpopulation.

Future longitudinal studies should extend the current evaluation in several directions. The administration of the MMSE and MoCA test at the same visit occasion should be integrated within a psycho-social questionnaire-based evaluation of subject’s needs, support network, medical condition, and daily life abilities to assess subject’s attitude towards successful ageing. If in doubt, the Medical Commission for the renewals should request results of practical sessions with driving simulators; although collected on a case-by-case basis, future studies on fragile subpopulations of major interest could start from these additional examinations. Alternatively, a record linkage procedure with data from the Department of Motor Vehicles of Milan would provide access to car injuries for this large population of oldest old subjects. This would allow to test our hypothesis of long-term memory being the most important cognitive domain to be targeted for assessing the presence of strategic abilities in safe driving.

Within a unified approach where cognitive reserve and successful ageing represent the key theoretical criteria, our analysis and future extensions highlight that it is indeed possible to use information on socio-demographic characteristics and cognitive performance to support the identification of older subjects still able to drive and to overcome socio-cultural barriers preventing a healthy aging. Well-being and a high quality of life in oldest old subjects would require them to maintain complex abilities, including driving, that we have targeted with disentangling overall cognitive performance into single domains with the necessary statistical power.

## Supplementary information

**Additional file 1.** “Socio-demographic characteristics and cognitive performance in oldest old subjects asking for driving license renewal”. Additional information on study design, data collection, and results.

## Data Availability

The datasets generated and/or analyzed during the current study are not publicly available due to a formal agreement between University of Milan and ATS Milano, but are available from the corresponding author on reasonable request.

## Abbreviations

ATS Milano: Agenzia di Tutela della Salute della Città Metropolitana di Milano
MCI: Mild cognitive impairment
MMSE: Mini mental state examination
MoCA: Montreal cognitive assessment
