# Supplementary material for: Socio-demographic characteristics and cognitive performance in oldest old subjects asking for driving license renewal
Source: BMC Geriatr. 2020 Jul 11;20:241. doi: 10.1186/s12877-020-01637-1 (PMC7353803; doi:10.1186/s12877-020-01637-1)
Supplement: Supplementary file 1 — Additional file 1. “Socio-demographic characteristics and cognitive performance in oldest old subjects asking for driving license renewal”. Additional information on study design, data collection, and results. [file 12877_2020_1637_MOESM1_ESM.pdf]

Additional file: “Socio-demographic characteristics and cognitive performance in oldest old subjects asking for driving license renewal”

1. Materials and methods

1.1 Study design

Since May 2011, the Legal Medicine Section of the University of Milan collected information on a brief anamnesis and cognitive performance from either the MMSE or the MoCA tests for any subjects over 80 years that was sent there for driving license renewal by the Local Medical Commission of the Agency for Health Protection of Milan (Agenzia di Tutela della Salute della Città Metropolitana di Milano - ATS Milano), Milan, Italy. Indeed, University of Milan was involved in a broader expert-based program of assessment of cognitive performance of oldest old subjects asking for driving license renewal established by ATS Milano to accomplish the more recent Italian regulation (Law 120 "Provisions on road safety", 2010) in providing a comprehensive evaluation of the ability to guide in oldest old subjects. Collected information was then sent back to the ATS Milano, where their dedicated Medical Commission decided in favor of driving license renewal or not (as established by Law 120 "Provisions on road safety", 2010). A special protocol was followed when either the test score was below the normality cut-off or other relevant medical conditions were discovered/reported during the examination. At any driving license expiration and subsequent renewal request of subjects >=80 years old, the Local Medical Commission asks the necessary cognitive

performance re-evaluation, subject to the presence of the other health requirements.

Any screening of cognitive assessment was administered by one in a group of expert psychologists from the University of Milan who filled in a hard document during the interview, and reviewed results at a later stage.

1.2 Selection of variables

The MMSE is based on 30 items that explore the following six domains: orientation to time and place (i.e. visuospatial orientation), short-term memory, long-term memory, attention and calculation, language (in the comprehension, repetition, naming, reading, and writing components), and constructive apraxia. The total score ranges from 0 to 30 and a score of 24 or higher indicates normal cognitive functions. The MMSE is a widely used brief screening instrument for dementia and it is also commonly used as a proxy for staging of AD [1], but it is not sensitive enough to distinguish MCI cases from healthy subjects. This depends on its substantial lack of complexity and assessment of executive function [2].

As compared to the MMSE, the MoCA is a more sensitive, but less specific, test [3]. It may predict dementia in people with MCI, and, because it tests for executive function, it is useful for people with scores of 26 or higher on the MMSE. Finally, it has been shown to identify cognitive problems in people with Parkinson's disease. It is still based on 30 items and its total score ranges from 0 to 30, but it has a longer time of administration (10-12 minutes). The items investigates visuospatial and executive functioning, animal naming, language (repetition and fluency components), attention, abstraction, short and

long-term memory, and orientation. An overall score equal to (or greater than) 26 indicates normal cognitive functioning.

To improve comparability between the single domain scores of the two tests, we modified a few single domain definitions and names in the following way:

1. MoCA - **visuospatial and executive functioning**: this domain was divided into two smaller ones: **constructive apraxia** (from Trail Making Test and cube drawing) and **visuospatial functioning** (from the Clock Drawing Test); this allows to compare results on constructive apraxia from the two tests;
2. MoCA – (animal) **naming**: this domain was added to the **language** one, as these skills contributed to the same item score in the MMSE test;
3. MoCA – **attention**: we called this domain **attention and calculation** in the current paper, because calculation is assessed within the attention domain in the MoCA test;
4. MoCA – **orientation**: we called this domain **visuospatial orientation**, because orientation to both time and place is assessed within this domain in the MoCA test.

Between February and May 2017, socio-demographic characteristics and test scores were entered in a database (on a visit base) by one dedicated student.

1.3 Selection of subjects

From May 2011 to March 2017, 4840 driving license renewal visits were carried out at the University of Milan. Seventy-two visits were discarded, as subjects were younger than 80 years of age at the first testing occasion.

We carried out several checks on the remaining 4768 visits. First, we solved any inconsistencies on subject's identification across subsequent visits. In case of subjects with the same first and last names, but different dates of birth and years of education across visits, we re-checked the original documents and we correctly identified those subjects as different subjects giving them different identification codes. In case of subjects with slightly different first or last names (including presence of the middle name or not), but the same years of education, sex, and date of birth across visits, we imputed the same identification codes across visits. In case of subjects with the same (first and last) name, but slightly different information on years of education, we imputed the subject-specific median of the variable 'years of education' across visits. Finally, in presence of the same (first and last) name, but inconsistent information on age across visits, we-recalculated age as date of interview – date of birth.

Checks were also made on minimum and maximum values of age, years of education, total and single domain scores (see Table 1) and inconsistencies were corrected.

Overall, there were 3392 (71.14%) first visits, 945 (19.82%) second visits, 307 (6.44%) third visits, 92 (1.93%) fourth visits, 29 (0.61%) fifth visits, 2 (0.04%) sixth visits, and only 1 (0.02%) seventh visit. In addition, the MMSE was administered in 3764 (78,94%) visits and the MoCA was used in 1004

(21,06%) visits. The joint distribution of the number of visits and the type of test administered was provided in **eTable 3**.

In the current paper, we based our analysis on the 3378 first visits where the MMSE test was administered (99.59% of the first visits, 89.74% of the MMSE tests administered) and on the 863 second visits where the MoCA was administered (91.32% of the second visits, 85.96% of the MoCA tests administered). This gave a total of 4241 visits used for the analysis.

1.4 Statistical analysis

Ordinary least-squares linear regression models were used to assess the relationship between socio-demographic characteristics and total (or single domain) scores, with test score being the dependent variable, and socio-demographic characteristics the independent variables. For each socio-demographic characteristic and test, we ran a simple linear regression model (i.e. one socio-demographic characteristic at a time), followed by a multiple model including two socio-demographic characteristics, and by a model including the three socio-demographic characteristics simultaneously. Age and years of education were considered as either continuous or categorical (ordinal) variables.

We also fitted the more complex two- and three-way interactions models, including two-way and two- and three-way interactions between age, sex, and years of education in categories, respectively. Model selection was carried out through likelihood ratio tests. P-values were two-sided.

Calculations were carried out using the open-source statistical computing environment R [4].

2. Results

eTable 4 shows some descriptive statistics representing the distribution of total and single domain scores for the MMSE and MoCA tests in our population. Single domain scores generally covered the entire range of possible values; however, for both tests, the minimum values for the easiest visuospatial orientation and language domains were higher than 0 and were even higher for the MMSE test, as compared to the MoCA: 3 out of 10 vs. 1 out of 6 for the former, and 4 out of 8 vs. 1 out of 6 for the latter domain. For most single domains of the MMSE test, the first quartile of the distribution was equal to the maximum value of the score; this implies that 75% of the sample reached the highest values of single domain scores in the MMSE test. A similar effect, although weaker, was evident for the single domains of the MoCA test: 50% of the sample reached the maximum values of single domain scores. Notably, for both tests, the long-term memory domain showed a sparser distribution. In addition, given the same 0-30 set of possible values for both tests, the minimum value for the total MMSE test score was 17, as compared to 7 for the total MoCA score. Median and mean values of total and single domain scores were similar and this pointed to symmetric distributions for the dependent variables under consideration.

References

1. Vertesi A, Lever JA, Molloy DW, Sanderson B, Tuttle I, Pokoradi L, Principi E: **Standardized Mini-Mental State Examination. Use and interpretation**. *Can Fam Physician* 2001, **47**:2018-2023.
2. Tombaugh TN, McIntyre NJ: **The mini-mental state examination: a comprehensive review**. *J Am Geriatr Soc* 1992, **40**(9):922-935.
3. Nasreddine ZS, Phillips NA, Bedirian V, Charbonneau S, Whitehead V, Collin I, Cummings JL, Chertkow H: **The Montreal Cognitive Assessment, MoCA: a brief screening tool for mild cognitive impairment**. *J Am Geriatr Soc* 2005, **53**(4):695-699.
4. **R Development Core Team. R: A Language and Environment for Statistical Computing 2019 Vienna, Austria: R Foundation for Statistical Computing, Available at: http:// www.R-project.org (26 May 2019, date last accessed)**
5. Wagner JT, Muri RM, Nef T, Mosimann UP: **Cognition and driving in older persons**. *Swiss Med Wkly* 2011, **140**:w13136.
6. Michon JA: **A critical view of driver behavior models: what do we know, what should we do?** In: *Human behavior and traffic safety.* Springer; 1985: 485-524.

eTable 1. Definition of single cognitive functions implied in driving, as identified in the paper by Wagner and coauthors [5].

**Cognitive function**	**Definition, with some examples of driver behaviors**
Visual attention	process that selects visual stimuli based on their spatial location
Visual perception	perception and correct interpretation of visual information
Executive function	ability to respond to novel situations in an adaptive manner, including volition, planning, anticipation and effective performance
Episodic memory	registration, acquisition and encoding of information, such as knowing where to find the car keys
Semantic memory	knowledge about the world, for example the meaning of colors of a traffic light
Procedural memory	ability to use a learned skill in an unconscious, automatic way, such as technically handling a car, for example starting the engine or switching gears

eTable 2. Classification of driving abilities and cognitive domains, as measured from the Mini Mental State Examination and the Montreal Cognitive Assessment tests, in terms of the three hierarchical levels of cognitive control of driving suggested within the Michon model [6].

		**Driver Behavior†,‡,§**	
	**Strategic Level**	**Tactical Level**	**Operational Level**
**Driving ability**	Driving with a copilot, planning the trip before driving, hitting the traffic, avoiding dangerous driving situations	Choosing direction, speed adaptation, anticipatory behavior	Steering, braking, using the vehicle controls, shifting gears
**Cognitive functions measured by MMSE (range of possible score values)**	Short-term memory† *(0-3)*<br>*Long-term memory (0-3)*<br>*Language (0-8)*	Short-term memory† *(0-3)*<br>*Attention and calculation (0-5)*	*Visuospatial orientation (0-10)*<br>*Constructive apraxia (0-1)*
**Cognitive functions measured by MoCA (range of possible score values)**	*Long-term memory (0-5)*<br>Abstraction‡ *(0-2)*<br>*Language (0-6)*	*Attention and calculation (0-6)*	*Visuospatial orientation (0-6)*<br>Visuospatial functioning‡ *(0-3)*<br>*Constructive apraxia (0-2)*

ABBREVIATIONS: MMSE: Mini Mental State Examination test; MoCA: Montreal Cognitive Assessment test.

†Domains available in the MMSE test only.

‡ Domains available in the MoCA test only.

§ Domains common to both tests were indicated in italics.

eTable 3. Joint raw (%) distribution of the number of visits and of the type of test (Mini Mental State Examination or Montreal Cognitive Assessment) used.

Number of visits	MMSE test	MoCA test
1	3378 (99.59%)	14 (0.41%)
2	82 (8.68%)	863 (91.32%)
3	247 (80.46%)	60 (19.54%)
4	42 (45.65%)	50 (54.35%)
5	13 (44.83%)	16 (55.17%)
6	2 (100%)	0 (0%)
7	0 (0%)	1 (100%)

ABBREVIATIONS: MMSE: Mini Mental State Examination test; MoCA: Montreal Cognitive Assessment test.

eTable 4. Descriptive statistics summarizing distributions of total and single domain scores for the Mini Mental State Examination and the Montreal Cognitive Assessment tests†.

	MMSE test score						MoCA test score					
	**Min**	**1st quartile**	**Median**	**Mean**	**3rd quartile**	**Max**	**Min**	**1st quartile**	**Median**	**Mean**	**3rd quartile**	**Max**
Domain												
Visuospatial orientation	3.00	10.00	10.00	9.71	10.00	10.00	1.00	6.00	6.00	5.90	6.00	6.00
Short-term memory	0.00	3.00	3.00	2.97	3.00	3.00	-	-	-	-	-	-
Attention and calculation	0.00	5.00	5.00	4.85	5.00	5.00	0.00	5.00	6.00	5.41	6.00	6.00
Long-term memory	0.00	1.00	2.00	2.03	3.00	3.00	0.00	1.00	2.00	2.01	3.00	5.00
Language	4.00	8.00	8.00	7.79	8.00	8.00	1.00	5.00	6.00	5.35	6.00	6.00
Constructive apraxia	0.00	1.00	1.00	0.93	1.00	1.00	0.00	1.00	2.00	1.53	2.00	2.00
Visuospatial functioning	-	-	-	-	-	-	0.00	2.00	3.00	2.63	3.00	3.00

Abstraction	-	-	-	-	-	-	0.00	1.00	2.00	1.72	2.00	2.00
Total (raw) score	17.00	28.00	29.00	28.28	30.00	30.00	7.00	23.00	25.00	24.54	27.00	30.00

ABBREVIATIONS: MMSE: Mini Mental State Examination test; MoCA: Montreal Cognitive Assessment test.

†Domains were presented in the order in which the MMSE test assessed them.
